# Eelgrass Sediment Microbiome as a Nitrous Oxide Sink in Brackish Lake Akkeshi, Japan

**DOI:** 10.1264/jsme2.ME18103

**Published:** 2018-12-01

**Authors:** Tatsunori Nakagawa, Yuki Tsuchiya, Shingo Ueda, Manabu Fukui, Reiji Takahashi

**Affiliations:** 1 College of Bioresource Sciences, Nihon University 1866 Kameino, Fujisawa, 252–0880 Japan; 2 Institute of Low Temperature Science, Hokkaido University Kita-19, Nishi-8, Kita-ku, Sapporo, 060–0819 Japan

**Keywords:** nitrous oxide-reducing microbiome, *nosZ*, *amoA*, eelgrass sediments, sulfur-oxidizing *Gammaproteobacteria*, *Flavobacteriia*

## Abstract

Nitrous oxide (N_2_O) is a powerful greenhouse gas; however, limited information is currently available on the microbiomes involved in its sink and source in seagrass meadow sediments. Using laboratory incubations, a quantitative PCR (qPCR) analysis of N_2_O reductase (*nosZ*) and ammonia monooxygenase subunit A (*amoA*) genes, and a metagenome analysis based on the *nosZ* gene, we investigated the abundance of N_2_O-reducing microorganisms and ammonia-oxidizing prokaryotes as well as the community compositions of N_2_O-reducing microorganisms in *in situ* and cultivated sediments in the non-eelgrass and eelgrass zones of Lake Akkeshi, Japan. Laboratory incubations showed that N_2_O was reduced by eelgrass sediments and emitted by non-eelgrass sediments. qPCR analyses revealed that the abundance of *nosZ* gene clade II in both sediments before and after the incubation as higher in the eelgrass zone than in the non-eelgrass zone. In contrast, the abundance of ammonia-oxidizing archaeal *amoA* genes increased after incubations in the non-eelgrass zone only. Metagenome analyses of *nosZ* genes revealed that the lineages *Dechloromonas-Magnetospirillum-Thiocapsa* and *Bacteroidetes* (*Flavobacteriia*) within *nosZ* gene clade II were the main populations in the N_2_O-reducing microbiome in the *in situ* sediments of eelgrass zones. Sulfur-oxidizing *Gammaproteobacteria* within *nosZ* gene clade II dominated in the lineage *Dechloromonas-Magnetospirillum-Thiocapsa*. *Alphaproteobacteria* within *nosZ* gene clade I were predominant in both zones. The proportions of *Epsilonproteobacteria* within *nosZ* gene clade II increased after incubations in the eelgrass zone microcosm supplemented with N_2_O only. Collectively, these results suggest that the N_2_O-reducing microbiome in eelgrass meadows is largely responsible for coastal N_2_O mitigation.

Nitrous oxide (N_2_O) is a powerful, long-lived greenhouse gas (45) and causes the depletion of the stratospheric ozone layer (54). In natural ecosystems, N_2_O is predominantly produced through the microbial processes of denitrification, nitrification, and nitrifier-denitrification (27, 72, 83). Among these mechanisms, coastal and estuarial N_2_O sources are assumed to be mainly due to sediment denitrification (46, 47). N_2_O emissions from an open ocean have recently been attributed to nitrification by ammonia-oxidizing archaea (AOA) (40, 61). However, limited information is currently available on the relationship between N_2_O emissions and nitrifiers in seagrass sediments. Moreover, the consumption of N_2_O was previously reported in sediments of eelgrass (*Zostera marina*) meadows (47). Considerable sediment N_2_O uptake has recently been reported in pristine shallow coastal ecosystems (20, 41), and rapid N_2_O reduction has been discovered in the suboxic ocean (4).

Denitrification is the sequential reaction of nitrate to the gaseous products N_2_O and/or nitrogen gas (N_2_) *via* nitrite (NO_2_^−^) and nitric oxide (NO) (NO_3_^−^→NO_2_^−^→NO→N_2_O→N_2_). Z-type N_2_O reductase (NosZ) is a key enzyme that catalyzes the reduction of N_2_O to N_2_ during denitrification under sufficiently low molecular oxygen conditions (11, 34). Therefore, the step of N_2_O reduction by the enzyme NosZ is a major process that influences N_2_O flux to the atmosphere (72, 84). Many prokaryotes, including more than 60 genera of bacteria, have the ability to denitrify heterotrophically (65). Autotrophic denitrifiers are also able to utilize nitrate (NO_3_^−^) and/or NO_2_^−^ as the electron acceptor and reduce N_2_O to N_2_ using the enzyme NosZ (64). Some autotrophic denitrifying sulfur oxidizers have the *nosZ* gene on the whole genome, such as *Thiobacillus denitrificans* and *Sulfuritalea hydrogenivorans* within *Betaproteobacteria* (5, 33), *Sedimenticola thiotaurini* within *Gammaproteobacteria* (19), and *Sulfurimonas denitrificans* within *Epsilonproteobacteria* (67). Chemolithoautotrophic denitrifiers within *Gammaproteobacteria* and *Epsilonproteobacteria* play an important role in the nitrogen cycle in the oxygen minimum zone in the ocean (37). *nosZ* genes have recently been classified into two phylogenetically distinct NosZ clades: the first encodes typical NosZ proteins, now designated as *nosZ* gene clade I, while the other encodes atypical NosZ proteins, now designated as *nosZ* gene clade II, including the denitrifying members of *Gammaproteobacteria*, *Epsilonproteobacteria*, and the phylum *Bacteroidetes* as well as the non-denitrifying microorganisms of genera such as *Anaeromyxobacter*, *Dyadobacter*, and *Ignavibacterium* (29, 60). PCR and metagenomic analyses based on the *nosZ* gene revealed that *nosZ* gene clade II is more abundant and widespread than *nosZ* gene clade I in several environments such as soil (29, 30, 43, 51), wastewater treatment plants (29), marine water (70), and marine sediments (3, 56, 79). Furthermore, previous studies demonstrated that non-denitrifying N_2_O-reducing bacteria within *nosZ* gene clade II played an important role in the consumption of N_2_O within soil (14, 52, 81). However, limited information is currently available on the distribution and community structure of microbiomes with the capacity to reduce N_2_O in eelgrass sediments.

Seagrass meadows provide the stabilization of sediment, a habitat for ecologically diverse and productive ecosystems that reduce the exposure of humans, fishes, and invertebrates to bacterial pathogens in coastal environments (16, 38). Sediments inhabited by seagrasses are generally anoxic due to sulfide produced by sulfate-reducing bacteria that utilize sulfate as an electron acceptor for the mineralization of organic matter accumulated by seagrasses (9). Molecular ecological studies based on 16S rDNA previously revealed the predominance of sulfur-oxidizing bacteria (SOB) within *Gammaproteobacteria* and/or *Epsilonproteobacteria* together with sulfate-reducing bacteria in seagrass sediments (10, 12, 17, 73). Some SOB within *Gammaproteobacteria* and/or *Epsilonproteobacteria* possess the *nosZ* gene (22, 28). Since high denitrification rates have been reported within the surface of seagrass sediments (8), we hypothesized that SOB possessing the *nosZ* gene may be responsible for the N_2_O sink in seagrass sediments alongside their role in sulfide detoxification for seagrasses growing in sulfidic sediments (24, 75).

To test this hypothesis, we characterized the microbiomes responsible for N_2_O reduction in eelgrass meadow sediments using the high-throughput sequencing of the *nosZ* gene and quantitative PCR (qPCR) analyses of *nosZ* and ammonia monooxygenase subunit A (*amoA*) genes. Since PCR primers for *nosZ* gene clade II have limitations for investigating the diversities of the genera *Anaeromyxobacter* and *Sulfurimonas* and the phylum *Bacteroidetes* due to PCR bias (29), we examined the community structures of the N_2_O-reducing microbiome using shotgun metagenomic analyses based on *nosZ* gene clades I and II. The community structures of microbiomes in sediments were compared between non-eelgrass and eelgrass zones. Laboratory incubations of sediment microcosms were also conducted for sediments from both zones to elucidate the relationship between the community structures of the N_2_O-reducing microbiome and N_2_O sink. Since few studies have investigated the influence of nitrogen fertilizers on the coastal areas, such as the sharp increase in (NH_4_)_2_SO_4_ due to increased precipitation, we examined the influence of ammonium on nitrification and N_2_O production in non-eelgrass and eelgrass zone sediments.

## Materials and Methods

### Study area and sampling

The study area was Lake Akkeshi, a brackish lake located in Hokkaido, Japan. Most of the lake (31.8 km^2^) is covered with the eelgrass *Zostera marina* (80). Sediment core samples were obtained at two different positions, a non-eelgrass zone (43°03′54″N, 144°51′36″E) (*n*=1) (Fig. S1A and C) and an eelgrass zone (43°03′18″N, 144°53′24″E) (*n*=1) (Fig. S1B and D), by a diver using a plastic corer (8 cm in diameter and 50 cm in length) on 28 July, 2015. A sample of the surface water was also obtained in a 1-L sterilized plastic bottle at the two different zones described above. The temperature, pH, dissolved oxygen (DO), concentrations of NO_3_^−^ and sulfate (SO_4_^2−^) in surface water, and water depth in the non-eelgrass zone were 18.2°C, pH 7.6, DO 6.2 mg L^−1^, 4.1 μM and 17.3 mM in surface water, and 0.5 m, respectively. The eelgrass zone had a temperature of 19.6°C, pH of 7.5, DO of 6.5 mg L^−1^, 3.5 μM NO_3_^−^, 19.3 mM SO_4_^2−^, and water depth of 1.0 m. A 0.0–4.0-cm layer of sediment (Fig. S1E) was sliced from the core using a stainless steel corer (7.0 cm in diameter and 4.0 cm in length). While the color of the sediment obtained from the non-eelgrass zone was dark brown, that from the eelgrass zone was black and sulfidic (Fig. S1F and G). The sediments collected were placed into sterilized 50-mL plastic tubes. The concentrations of NO_3_^−^ and SO_4_^2−^ in the pore water of sediments were 8.1 μM and 20.2 mM in the non-eelgrass zone and 5.2 μM and 20.9 mM in the eelgrass zone, respectively. Samples were transferred to the laboratory in an ice-cooled box within 3 d. Sediments were centrifuged in the sterile 50-mL plastic tubes (5,800×*g*, 4°C, 10 min) and then mixed well after the supernatant had been discarded. Sediments for the incubation test were kept on ice until incubation experiments. Sediments for DNA extraction were stored at −80°C. One liter of seawater from the two different zones was filtered using Nalgene Rapid-Flow Filters (pore size, 0.2 μm; Thermo Fisher Scientific, Waltham, MA, USA).

### Cultivation of sediment microcosms and N_2_O analysis

Sediment incubation experiments (three biological replicates) were performed for the non-eelgrass zone and eelgrass zone. Fifty-milliliter serum bottles (GL Sciences, Tokyo, Japan) containing 5 g of sediments and 15 mL of filter-sterilized seawater (Fig. S1F and G) were closed with black butyl rubber stoppers (Nichiden-Rika Glass, Kobe, Japan), capped with an aluminum crimp seal (GL Sciences), and then incubated at 20°C for 7 d in the dark. The bottles were shaken by hand for a few second once a day during the 7-d incubation (except on day 6 of the incubation). Three treatments were prepared for two different zones: one as a control (not amended) designated with the sample names N1 for the non-eelgrass zone and E1 for the eelgrass zone, one treated with 3.2 mM NH_4_Cl seawater (N2 for the non-eelgrass zone and E2 for the eelgrass zone) to enhance the activity of ammonia oxidizers, and one spiked with 0.3 mL of 99.5% N_2_O (N3 for the non-eelgrass zone and E3 for the eelgrass zone) in order to confirm differences in net N_2_O absorption between the non-eelgrass and eelgrass zones. Each treatment was conducted in triplicate, giving a total of nine bottles in each zone. The gas in the headspace of the bottle after day 7 of the incubation was withdrawn *via* the closed butyl rubber stopper using a Pressure-Lok precision analytical syringe (VICI Precision Sampling, Baton Rouge, LA, USA) for the N_2_O analysis. N_2_O concentrations were measured with a gas chromatograph equipped with an electron capture detector (GC-14B; Shimadzu, Kyoto, Japan). Sediment and seawater in serum bottles after day 8 of the incubation were centrifuged in the sterile 50-mL plastic tubes (5,800×*g*, 4°C, 10 min) and then stored at −80°C after the supernatant had been discarded.

### Nucleic acid extraction

Each wet sediment (~0.5 g) before (*in situ*) and after the incubation was added to a plastic tube containing lysis solutions and beads in ISOIL for the Beads Beating Kit (Nippon Gene, Toyama, Japan), mixed vigorously for 45 s, and then incubated at 65°C for 1 h. Nucleic acids were extracted according to the manufacturer’s protocol, and extracted nucleic acids in 20 μL of Tris-EDTA (TE) buffer were then stored at −20°C. Twenty-four DNA samples were extracted from the incubated sediments (N1, N2, N3, E1, E2, and E3) and *in situ* sediments were designated with the sample names Ni for the non-eelgrass zone and Ei for the eelgrass zone.

### qPCR of *nosZ* and *amoA* genes

Regarding the quantification of *nosZ* and *amoA* gene copies, each DNA solution extracted from sediments before (*in situ*) and after the incubation was quantified by real-time PCR in a CFX96 Real-Time System (Bio-Rad, Hercules, CA, USA) using the SYBR Premix Ex Taq (Tli RNase H Plus) kit (TaKaRa Bio, Kusatsu, Japan). Twenty-three of the DNA samples were used for qPCR: duplicate DNA samples from N3 due to the loss of samples, and triplicate DNA samples from the remaining sediment samples. The following detection primer sets were used: nosZ2F and nosZ2R for *nosZ* gene clade I (26), modified nosZ-II-Fn (5′-CTN GGN CCN YTK CAY AC-3′) and nosZ-II-Rn (5′-GCN GAR CAR AAN TCB GTR C-3′) for *nosZ* gene clade II (29), GenAOAF and GenAOAR for the AOA *amoA* gene (44), and *amoA*-1F and *amoA*-2R for the beta-proteobacterial ammonia-oxidizing bacteria (AOB) *amoA* gene (57). Standard curves were generated from a plasmid containing each of the following cloned genes: the *nosZ* clade I gene fragment of *Pseudomonas stutzeri* NBRC14165 amplified with the PCR primers nosZ_1F (15) and nosZ2R for *nosZ* gene clade I (26), the *nosZ* clade II gene fragment of the environmental clone G3H008 amplified with the PCR primers nosZ-II-Fn and nosZ-II-Rn (Fig. S2), the AOA *amoA* gene fragment of *Nitrosopumilus* sp. NM25 amplified with the PCR primers CrenAMO_F and CrenMO_R (23), and the AOB *amoA* gene fragment of *Nitrosomonas stercoris* KYUHI-S^T^ amplified with the PCR primers *amoA*-1F and *amoA*-2R. Each reaction was performed in a volume of 25 μL containing 2 μL of diluted DNA solution (one fiftieth of template DNA), 0.2 μM of each primer (1.0 μM of each primer only for *nosZ* gene clade I), and 12.5 μL of SYBR Premix Ex Taq (Tli RNase H Plus). Cycling conditions were as follows: for *nosZ* clade I (26), an initial denaturation step at 95°C for 3 min, followed by 6 cycles at 95°C for 15 s, 65°C for 30 s, and 72°C for 30 s, and then 40 cycles at 95°C for 15 s, 65°C for 15 s, 72°C for 30 s, and 80°C for 15 s. Fluorescence intensity was measured at 80°C. Regarding *nosZ* clade II (29), cycling conditions were an initial denaturation step at 95°C for 3 min, followed by 40 cycles at 95°C for 10 s, 60°C for 30 s, 72°C for 30 s, and 80°C for 30 s. Fluorescence intensity was measured at 80°C. Cycling conditions for AOA *amoA* (44) were an initial denaturation step at 95°C for 3 min, followed by 40 cycles at 95°C for 10 s, 55°C for 30 s, 72°C for 30 s, and 80°C for 1 s. Fluorescence intensity was measured at 80°C. Regarding AOB *amoA* (2), cycling conditions were an initial denaturation step at 95°C for 3 min, followed by 40 cycles at 95°C for 10 s, 57°C for 30 s, 72°C for 30 s, and 78°C for 1 s. Fluorescence intensity was measured at 78°C. After each run, the amplicon was visualized on an agarose gel to confirm the specific product bands of the expected size. Efficiencies for *nosZ* clade I, clade II, AOA *amoA*, and AOB *amoA* amplification were estimated at 62, 62, 108, and 82%, with *R*^2^ of 0.963, 0.999, 0.980, and 0.998, respectively.

### Metagenomic library construction and sequencing of *nosZ* genes

The library for the shotgun metagenomic analysis for *nosZ* genes was created with the tagmentation-based Nextera DNA Library Prep Kit (Illumina, San Diego, CA, USA) according to the manufacturer’s protocol, and samples were then stored at −20°C. Twenty-one DNA samples were used for tagmentation: a single DNA sample from N1 due to the loss of samples, duplicate DNA samples from N3 due to the loss of samples, and triplicate DNA samples from the remaining sediment samples. The DNA quality of each library was verified by 2200 TapeStation (Agilent Technologies, Santa Clara, CA, USA), and quantified with the QubiT dsDNA HS assay kit and QubiT fluorometer (Life Technologies, Carlsbad, CA, USA). The sequencing of composite DNA samples was performed using the MiSeq V2 reagent kit (2×150 bp) on MiSeq (Illumina).

The initial quality filtering of paired-end reads was performed with MiSeq software version 2.5.0.5 (Illumina) to remove some reads with base calls below the threshold (Q30) and the trim sequences of tag and adapter. All paired-end Illumina reads were imported into CLC Genomic Workbench version 8.5.1 (CLCBio; QIAGEN, Aarhus, Denmark), and some reads that were shorter than 90 nucleotides were discarded from the libraries, resulting in 38,029,139 read numbers (Table S1). Due to the limitation of computer memory, 30% of the N1, N2, and N3 reads were used in subsequent analyses. To detect *nosZ* reads in the metagenomes derived from each sediment sample, publicly available NosZ amino acid references were downloaded from FunGene (http://fungene.cme.msu.edu) (18) of the Ribosomal Database Project (RDP) and the National Center for Biotechnology Information (NCBI), and then imported into the CLC Genomic Workbench. NosZ-encoding reads were identified by blastx (1) against NosZ amino acid references with an e-value threshold of 10^−15^. To exclude the non-NosZ amino acid sequences of uncultured bacteria with an e-value of more than 10^−15^ from the references, the amino acids of uncultured bacteria with an e-value of more than 10^−15^ in the NosZ amino acid references were aligned with archetype amino acid sequences using the CLUSTAL W program in MEGA version 7 (35). The phylogenetic tree was constructed by the maximum-likelihood method in MEGA7, resulting in 1,986 of *nosZ* gene reads (Table S1). Similarly, the numbers of nitric oxide reductase subunit B (*norB*) and *amoA* gene reads in the metagenomes were investigated as described in the supplementary information. Operational taxonomic units (OTUs) were defined as sequence groups based on the lineages constructed from the phylogenetic tree in order to compare *nosZ*-based diversity with a rarefaction analysis using Analytic Rarefaction 1.3 (https://strata.uga.edu/software/index.html).

### Statistical analysis

A one-way analysis of variance (ANOVA) was used to analyze the significance of differences in N_2_O concentrations and the abundance of *nosZ* and *amoA* genes before and after the incubation. Pearson’s product moment correlation (PPMC) analysis was performed to assess the relationship between the increased abundance of *amoA* genes and elevated concentrations of N_2_O in the headspace of bottles after the 7-d incubation. ANOVA and PPMC analyses were performed with SPSS Statistics version 20 (IBM, Armonk, NY, USA).

### Nucleotide sequence accession number

All metagenomics reported in the present study were deposited in the DNA Data Bank Japan (DDBJ) Sequence Read Archive (DRA) under accession number DRA006867. The corresponding tables between the sample names and deposit IDs of *nosZ* gene reads on DRA006867 have been submitted to FigShare (http://dx.doi.org/10.6084/m9.figshare.6726428).

## Results

### N_2_O emissions and sink

**Fig. 1:**
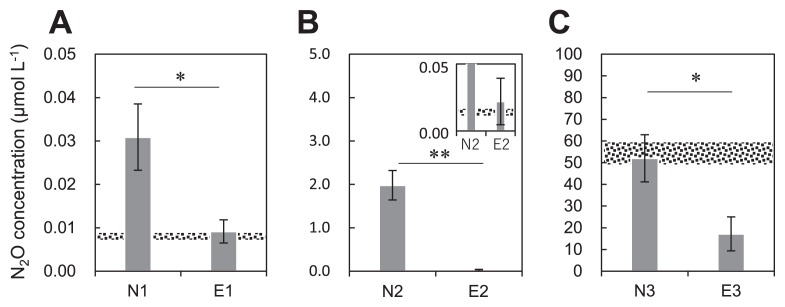
Concentrations of N_2_O in the headspace of bottles after a 7-d incubation. (**A**) Sediments incubated without NH_4_Cl or N_2_O in the non-eelgrass zone N1 and eelgrass zone E1. (**B**) Sediments incubated with NH_4_Cl in the non-eelgrass zone N2 and eelgrass zone E2. (**C**) Sediments incubated with N_2_O in the non-eelgrass zone N3 and eelgrass zone E3. Error bars indicate the standard deviation (*n*=3 biologically independent samples). * shows a significant difference (*, *P*<0.05; **, *P*<0.005). The gravel zone (at approximately 0.007 μmol L^−1^, and approximately 55 μmol L^−1^) shows the initial concentrations of N_2_O in the headspace of bottles.

N_2_O concentrations in the headspace after the incubation were significantly higher in non-eelgrass sediment bottles than in eelgrass sediment bottles (*P*=0.010) (Fig. 1A), indicating that N_2_O emissions were higher from non-eelgrass sediments than from eelgrass sediments. Even though ammonium was supplemented to sediments, the N_2_O concentration in eelgrass sediment bottles (0.022 μmol L^−1^) was similar to that in eelgrass sediment bottles without ammonium (0.009 μmol L^−1^) (Fig. 1B). In contrast, the N_2_O concentration in the headspace of non-eelgrass sediment was 90-fold higher than that in eelgrass sediment, implying low N_2_O emissions from eelgrass sediments. In addition, when a high concentration of N_2_O was injected into sediment bottles, N_2_O concentrations in the headspace after the incubation were significantly lower in eelgrass sediment bottles than in non-eelgrass sediment bottles (*P*=0.011) (Fig. 1C), suggesting that eelgrass sediments have the capability to absorb more N_2_O than non-eelgrass sediments.

### qPCR of *nosZ* and *amoA* genes

**Fig. 2:**
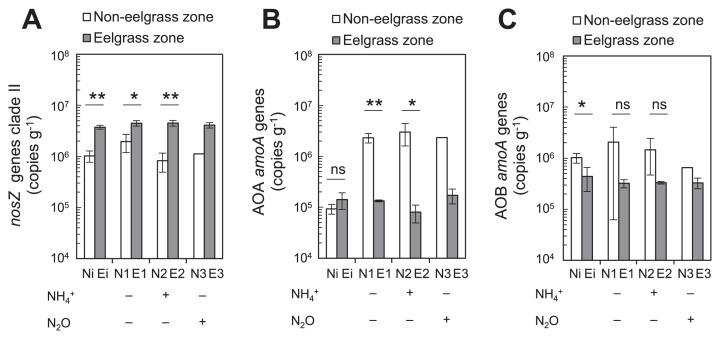
Abundance of *nosZ* gene clade II (**A**), AOA *amoA* genes (**B**), and AOB *amoA* genes (**C**) in *in situ* sediments and bottles after day 7 of an incubation of non-eelgrass and eelgrass zones. Error bars indicate the standard deviation (*n*=3 biologically independent samples). Only N3 is shown (*n*=2). * indicates a significant difference (*, *P*<0.05; **, *P*<0.005; ns, not significant).

The abundance of *nosZ* gene clade II in *in situ* sediments was significantly higher in eelgrass zone sediment Ei than in non-eelgrass zone sediment Ni (*P*=0.000) (Fig. 2A). Similarly, after the incubation, the abundance of *nosZ* gene clade II was higher in all types of bottles with eelgrass zone sediments E1, E2, and E3 than in all bottles with non-eelgrass zone sediments N1, N2, and N3. Although the target lengths of PCR products for *nosZ* gene clade I were detected by qPCR for all samples, the abundance of *nosZ* gene clade I was not elucidated because the fluorescent intensities of amplicons for the non-targeted region were as strong as those of the target PCR products after qPCR.

The abundance of the AOA *amoA* genes in incubated bottles with non-eelgrass zone sediments N1, N2, and N3 was approximately 10-fold higher than that in the *in situ* non-eelgrass zone sediment Ni (Fig. 2B). In contrast, no significant changes were observed in the abundance of AOA *amoA* genes between before and after the incubation among eelgrass sediment samples. In AOB *amoA* genes, no significant differences were noted between before and after the incubation for both sediment samples (Fig. 2C). The abundance of AOB *amoA* genes in *in situ* sediments from the non-eelgrass zone (Fig. 2C, Ni) was significantly higher than that of AOA *amoA* genes in *in situ* sediments from the non-eelgrass zone (Fig. 2B, Ni) (*P*=0.002). However, there was no significant change in the abundance of *amoA* genes between AOA (Fig. 2B, Ei) and AOB (Fig. 2C, Ei) in the eelgrass zone (*P*=0.082).

### *nosZ* gene metagenome

**Fig. 3:**
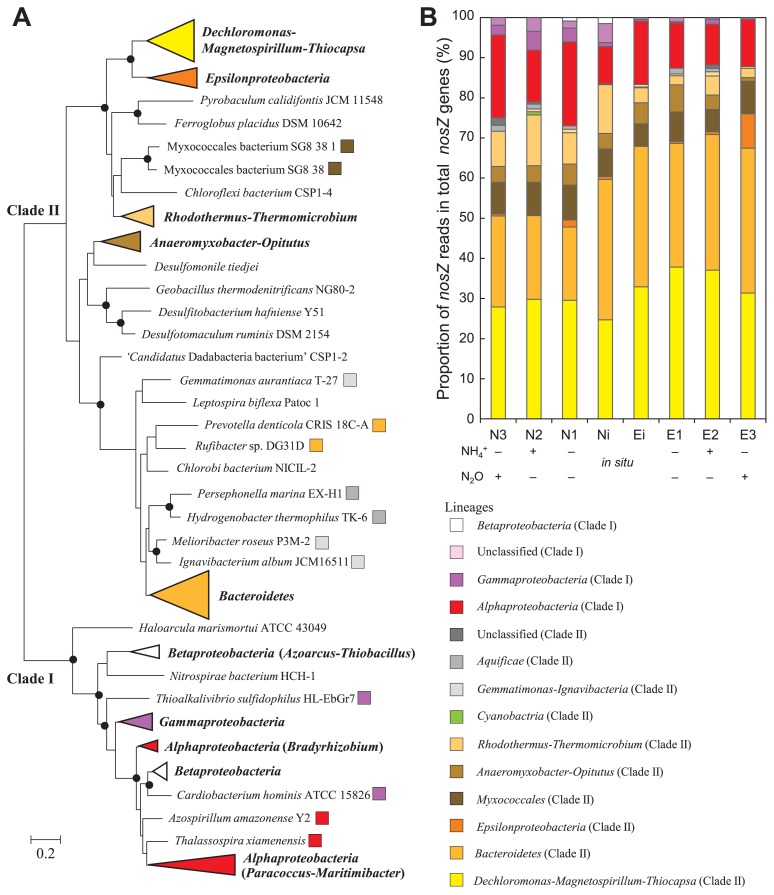
Bootstrapped maximum likelihood tree (**A**) and average relative abundance of *nosZ* genes (**B**). The tree was built from 138 archetype amino acid sequences. Branches with bootstrap support of more than 70% are revealed by closed circles. The scale bar represents an estimated sequence divergence of 20%. Data represent the mean of biologically independent samples (*n*=3). Only N1 is shown (*n*=1), and only N3 is shown (*n*=2).

Shotgun metagenomic analyses based on the *nosZ* gene revealed that *nosZ* gene reads mainly fell into the lineages *Dechloromonas*-*Magnetospirillum*-*Thiocapsa*, *Bacteroidetes*, *Myxococcales*, *Anaeromyxobacter*-*Opitutus*, *Rhodothermus*-*Thermomicrobium*, *Epsilonproteobacteria*, and *Gemmatimonadetes*-*Ignavibacteria* within *nosZ* gene clade II, and into the lineages *Alphaproteobacteria* and *Gammaproteobacteria* within *nosZ* gene clade I in the non-eelgrass and/or eelgrass zones (Fig. 3A). *nosZ* genes within *Bacteroidetes* and *Epsilonproteobacteria* were detected as predominant members using shotgun metagenomic sequencing (Fig. 3A and B) even though they were not detected by the cloning analysis (Fig. S2). The community structural proportions of *nosZ* gene clade II were approximately four-fold higher than those of *nosZ* gene clade I in *in situ* sediments for both zones (Fig. 3B). This result was consistent with previous findings on *nosZ* gene clade II (51, 56, 70). Furthermore, the numbers of *nosZ* gene reads were slightly higher than those of *norB* gene reads (Table S1).

In the eelgrass zone, the reads of *nosZ* gene clade II were occupied by the dominant members of the lineages *Dechloromonas*-*Magnetospirillum*-*Thiocapsa* (approximately 30%) and *Bacteroidetes* (approximately 30%) in *in situ* and incubated sediments (Fig. 3B, Ei, E1, E2, and E3). In the lineage *Dechloromonas*-*Magnetospirillum*-*Thiocapsa*, the reads of the sulfur-oxidizing gammaproteobacterial *nosZ* gene dominated (more than approximately 50%) in *in situ* and incubated sediments in the eelgrass zone (Table 1, Ei, E1, E2, and E3). The majority of sulfur-oxidizing *Gammaproteobacteria* were related to sulfur- and sulfide-oxidizing symbionts, such as *Thiolapillus brandeum* isolated from a hydrothermal vent (48), ‘*Candidatus* Thiodiazotropha endoloripes’ and ‘*Candidatus* Thiosymbion oneisti’ in seagrass sediments (53), the marine bivalve mollusk *Solemya velesiana* gill symbiont (58), and endosymbionts of the deep-sea tubeworms *Ridgeia piscesae* and *Tevnia jerichonana* (22). Further predominant *nosZ* gene reads were related to the giant sulfur-oxidizing bacterium ‘*Candidatus* Thiomargarita nelsonii’ (77) and anaerobic phototrophic nitrite oxidizer *Thiocapsa* sp. KS1 (25) in the *in situ* sediment of the eelgrass zone. In addition, the spiking of N_2_O into serum bottles (Table 1, E3 and N3) induced an increase in unclassified *nosZ* reads related to the cytochrome *c* N_2_O reductase (*c*Nos) of *Epsilonproteobacteria*, which dominated in the gill chamber epibiosis of the deep-sea shrimp (28).

In both zones, the lineage *Bacteroidetes* was dominated by *nosZ* reads related to marine *Flavobacteriia* (*Lutibacter*, *Seonamhaeicola*, *Maribacter*, *Arenibacter*, *Aquimarina*, *Cellulophaga*, *Bizionia*, and *Muricauda*), such as *Lutibacter profundi* (78), Flavobacteriales bacterium ALC-1 (7), and Flavobacteriaceae bacterium NORP142 (74) (Table 2). In N_2_O-supplemented sediment E3 in the eelgrass zone, in which the proportion of *Epsilonproteobacteria* increased (Fig. 3B), epsilonproteobacterial reads were related to *S. gotlandica* GD1 (36), *Sulfurospirillum multivorans* DSM 12446 (62), and *Arcobacter* spp. (13, 71). In the non-eelgrass and eelgrass zones, approximately 90% of alphaproteobacterial reads within *nosZ* gene clade I fell into the lineage *Azospirillum*-*Thalassospira*-*Maritimibacter*-*Paracoccus* (Fig. 3A); however, most alphaproteobacterial reads were related to uncultured bacteria. A rarefaction analysis showed that there was no significant difference in diversity among samples (Fig. S3).

## Discussion

### qPCR and shotgun metagenomic analyses

The qPCR analysis based on the *nosZ* gene revealed that the abundance of N_2_O-reducing microbes within *nosZ* gene clade II in *in situ* sediments of the eelgrass zone was 3.7-fold higher than that in the non-eelgrass zone (Fig. 2A). A previous study reported that PCR primers for *nosZ* gene clade II are limited by PCR bias (29). Although *Bacteroidetes* and *Epsilonproteobacteria* were detected in incubated sediment E3 (sample ID: aA8) with N_2_O in the eelgrass zone as predominant members using shotgun metagenomic sequencing (Fig. 3B), *nosZ* genes related to *Bacteroidetes* and *Epsilonproteobacteria* were not detected in the same sediment E3 (sample ID: aA8) by a PCR-dependent analysis based on the *nosZ* gene in the present study (Fig. S2). This result suggests an underestimation of the abundance of *nosZ* gene clade II within *Bacteroidetes* (*Flavobacteriia*) and *Epsilonproteobacteria*. However, a shotgun metagenomic analysis based on the *nosZ* gene not only detected sulfide-oxidizing *Gammaproteobacteria* within the lineage *Dechloromonas*-*Magnetospirillum*-*Thiocapsa*, but also successfully identified N_2_O-reducing microbes within *Flavobacteriia* and *Epsilonproteobacteria* from incubated sediment E3 of the eelgrass zone (Fig. 3). Therefore, a parallel analysis (qPCR and shotgun metagenomic sequencing) demonstrated that sulfide-oxidizing *Gammaproteobacteria* and marine *Flavobacteriia* were the dominant N_2_O-reducing microbes in *in situ* sediments of the eelgrass zone. However, a new qPCR primer set needs to be designed to accurately evaluate the enumeration of assemblages. In addition to the contribution of N_2_O-reducing microbes, since the numbers of *nosZ* reads detected were higher than those of *norB* reads in both *in situ* sediments (Table S1), the net N_2_O emission in *in situ* sediments also appears to have been suppressed by the lower abundance of microbes possessing nitric oxide reductase, which produces N_2_O.

### Sulfide scavengers for N_2_O reduction in sulfidic sediments

Laboratory incubation tests (Fig. 1) revealed N_2_O absorption by eelgrass sediments, which were black and sulfidic due to the vigorous sulfate-reducing activity of SRB in eelgrass zone (Fig. S1E). The continuous supply of hydrogen sulfide (H_2_S) from the bottom sediment and dissolved dioxygen (O_2_) from surface water appeared to be responsible for the growth of SOB within surface sediments in the eelgrass zone. The abundance of *nosZ* gene clade II in *in situ* sediments in the eelgrass zone was approximately four-fold higher than that in *in situ* non-eelgrass zone sediments (Fig. 2A). However, previous studies reported that sulfide inhibits the activity of N_2_O-reducing microbes (42, 50, 68). Furthermore, the reduction of N_2_O by *Anaeromyxobacter dehalogenans* was inhibited in laboratory culture medium amended with 0.2 mM sulfide (50). Sulfide concentrations may be reduced by SOB activity in seagrass sediments (24, 75). Therefore, SOB may act as sulfide scavengers, reducing sulfide stress for the NosZ enzyme activity of N_2_O-reducing microbes. In addition, the formation of FeS and FeS_2_ appeared to contribute to decreasing sulfide concentrations in eelgrass sediments.

### Production of N_2_O by nitrifiers

The production of N_2_O is attributed to nitrification and denitrification in marine sediments at low oxygen concentrations (31). In the non-eelgrass zone, laboratory incubation tests indicated net N_2_O emission from incubated sediments (Fig. 1A and B). The abundance of AOB was higher than that of AOA in *in situ* sediments of the non-eelgrass zone (Fig. 2B and C). This result supports recent findings indicating that AOB outnumbered AOA in estuary sediments influenced by human activity (39, 76, 82). However, the abundance of AOA markedly increased after the incubation for non-eelgrass sediments only (Fig. 2B). In the non-eelgrass zone, the increased concentration of N_2_O in the headspace of bottles and elevated copy numbers of AOA *amoA* genes after the incubation in both samples without ammonium and supplemented with ammonium (Fig. S4) showed positive correlations (*r*=0.922, *P*<0.01 for sediments incubated without NH_4_Cl; *r*=0.774, *P*=0.07 for sediments incubated with NH_4_Cl). AOA may grow under microaerobic conditions coupled with the production of N_2_O (55). Furthermore, dioxygen was presumed to be quickly depleted in the serum bottles. Therefore, N_2_O production by AOA and AOB appeared to contribute to N_2_O emissions in the non-eelgrass zone in addition to N_2_O production by denitrification. In contrast, net N_2_O emissions from sediments after the incubation were lower in the eelgrass zone than in the non-eelgrass zone (Fig. 1A and B). Furthermore, no significant increase in AOA or AOB *amoA* genes occurred after the incubation in eelgrass sediment bottles (Fig. 2B and C). Therefore, the production of N_2_O by nitrification appeared to be inhibited in incubated bottles from the eelgrass zone.

### Heterotrophic and autotrophic N_2_O reduction

*nosZ* genes within *Flavobacteriia* were detected from *in situ* sediments in the non-eelgrass and eelgrass zones (Fig. 3B). *Flavobacteriia* are one of the most abundant populations in aquatic environments (32) including seagrass sediments (12, 17, 73). They are proficient at degrading high-molecular-weight organic matter (32). Recent metagenomic analyses using next-generation sequencers demonstrated that some *Flavobacteriia* possess *nosZ* genes, suggesting their capacity to reduce N_2_O (7, 74, 78). Heterotrophic N_2_O reduction is preferable to gain energy, such as N_2_O reduction using acetate as the electron donor: 2 N_2_O+1.5 C_2_H_3_O_2_^−^→2 N_2_+HCO_3_^−^+1.5 H^+^ (Δ *G*°=−600 kJ per reaction) (37). N_2_O reduction may be one of the preferential reactions to yield energy for *Flavobacteriia* in the absence of dioxygen as an electron acceptor in sulfidic sediments.

Sulfur-oxidizing N_2_O-reducing microbes within *nosZ* gene clade II were one of the highest populations in sulfidic sediments of the eelgrass zone (Fig. 3B and Table 1). Autotrophic denitrification coupled with sulfide oxidation, 2 NO_3_^−^+5 HS^−^+7 H^+^→N_2_+5 S°+6 H_2_O (Δ *G*°=−1,260 kJ per reaction), is a favorable reaction for chemolithotrophs (37). The sulfur-oxidizing gammaproteobacterium *T. brandeum* (48) and sulfur-oxidizing epsilonproteobacterium *S. gotlandica* (36) have the ability to grow autotrophically with nitrate as an electron acceptor. Since *nosZ* genes related to *T. brandeum* and *S. gotlandica* were predominantly detected from sediments in the eelgrass zone in the present study, autotrophic denitrification by SOB also appears to be responsible for the N_2_O sink in sediments in the eelgrass zone. The highest rate of complete denitrification was reported at 40 m within the peak of H_2_S in the marine oxygen minimum zone off Peru, suggesting that autotrophic SOB reduced N_2_O with H_2_S in the oxygen minimum zone (63).

A previous study reported that the N_2_O consumption rates of the heterotrophic N_2_O reducers, *P. stutzeri*, *Shewanella loihica*, *Dechloromonas aromatica*, and *A. dehalogenans*, were 4.16, 0.446, 0.461, and 0.0171 μmol min^−1^ mg biomass^−1^, respectively (81). Similarly, the N_2_O consumption rate of the autotrophic N_2_O reducer *Thiohalorhabdus denitrificans* was 180–300 nmol min^−1^ mg protein^−1^ (69). Although no significant differences have been reported in N_2_O reduction rates between heterotrophic and autotrophic N_2_O reducers, the substrate affinity of clade II *nosZ* N_2_O reducers for N_2_O is generally higher than that of clade I *nosZ* N_2_O reducers (6, 59, 81). The *K_m_* value of soil *Flavobacteriia* sp. for N_2_O was 0.5 μM (6). Thus, the predominance of clade II *nosZ* N_2_O reducers in eelgrass sediments appears to be due to differences in affinity for N_2_O between clade I and clade II *nosZ* N_2_O reducers. Since the surface of eelgrass zone sediments was covered with dead leaves (Fig. S1D and E), the heterotrophic denitrifier *Flavobacteriia* may play an important role in the N_2_O sink with organic matter as an electron donor. However, soil *Flavobacteriia* sp. have been shown to produce N_2_O as oxygen tension increases (6). The *Flavobacteriia nosZ* phylotype was detected as the predominant member at the N_2_O peak within marine oxygen-deficient zones in the Eastern Tropical North Pacific (ETNP) (21). Further studies are needed to clarify whether heterotrophic and autotrophic N_2_O reducers contribute to the N_2_O sink in *in situ* eelgrass sediments.

## Conclusions

A shotgun metagenomic analysis based on the *nosZ* gene coupled with quantitative and physiological experiments suggested an N_2_O sink due to the N_2_O-reducing microbiome in sediments of the eelgrass zone. Seagrass meadows are widely distributed along coastal environments worldwide (66). Therefore, N_2_O-reducing microbiomes in seagrass meadows play an important role in the global nitrogen cycle, and have the potential to mitigate N_2_O from coastal environments worldwide. Future studies on the vertical distribution of N_2_O-reducing microbiomes coupled with the vertical dynamics of dissolved N_2_O, sulfide (HS^−^), NO_3_^−^, O_2_, ferrous (Fe^2+^), the oxidation-reduction potential, and stable isotopic composition of NO_3_^−^ (49) in sediments, and also on sulfur-oxidizing symbionts involved in N_2_O reduction are needed in order to provide a comprehensive understanding of the role of seagrass sediment microbiomes as an N_2_O sink. Since rapid precipitation is expected to increase, the effects of NO_3_^−^ outflow by nitrogen fertilizers on the production of N_2_O in eelgrass zone sediments warrant further study.

## Supplementary Information



## Figures and Tables

**Table 1:** Average relative taxonomic distribution of *nosZ* gene reads within the *Dechloromonas-Magnetospirillum-Thiocapsa* lineage at the genus level.

Genus of the lowest E-value (Accession numbers)	Sulfur-oxidizing bacteria	%^a^							

		N3	N2	N1	Ni	Ei	E1	E2	E3
*Thiolapillus brandeum* (WP_041065396)	S^b^	9	5	15	22	15	10	6	10
*Candidatus* Thiodiazotropha spp. (WP_069019464, WP_069128191)	S	9	16	6	8	14	10	12	13
*Solemya velesiana* gill symbiont (OOZ42363)	S	9	12	15	2	10	8	6	4
*Candidatus* Thiosymbion oneisti (WP_089723768)	S	9	5	15	14	8	9	15	4
*Candidatus* Thiomargarita spp. (OAD21140, KHD07088)	S	2	8	9	1	7	1	1	0
Gammaproteobacteria bacterium LUC14_002_19_P1 (OQX30387)	S	9	8	6	4	5	2	0	4
*Thiocapsa* spp. (EGV19470, CRI65417)	S	0	2	0	5	4	3	4	4
Gammaproteobacteria bacterium LUC003_P10 (OQX42463)	S	9	6	6	1	4	4	6	10
endosymbiont of *Ridgeia piscesae* (WP_060528325, KRT60036)	S	2	3	0	0	3	6	2	0
*Sulfuricella denitrificans* (WP_009206857)	S	0	0	0	0	1	6	1	0
*Thioploca ingrica* (BAP57181)	S	0	0	0	0	1	3	0	0
endosymbiont of *Tevnia jerichonana* (vent Tica) (EGW55450)	S	0	3	0	0	1	0	0	0
sulfur-oxidizing symbionts (WP_005958984)	S	4	0	3	0	0	1	0	0
*Sulfuritalea* sp. (AIC84793)	S	2	0	0	0	0	0	0	0
marine sediment metagenome (GAG54355)	nd^c^	2	5	0	10	4	10	8	19
*Sedimenticola* spp. (WP_029133254, WP_046859760)	nd	4	5	15	9	5	7	7	5
Gammaproteobacteria bacterium RIFOXYD12_FULL_61_37 (OGT89854)	nd	3	6	0	8	5	9	5	1
*Magnetospira* sp. (CCQ73153)	nd	0	2	0	2	4	0	0	2
*Magnetospirillum* spp. (CAM74903, CDK98645, BAE51890, KIM00076, OAN53142, OAN47899)	nd	3	1	0	2	2	0	4	0
Gammaproteobacteria bacterium HGW-Gammaproteobacteria-1 (PKM46105)	nd	0	1	3	1	2	4	0	0
*Thauera phenylacetica* (ENO96869)	nd	0	0	0	0	0	2	1	2
*Candidatus* Accumulibacter phosphatis (ACV36679)	nd	0	0	0	0	0	0	2	0
*Dechlorosoma aromatica* (AAZ46320)	nd	4	2	0	4	0	0	1	1
*Maritimibacter* sp. (WP_085526419)	nd	0	0	3	0	0	0	8	2
unclassified bacterium	nd	23	11	6	7	6	5	10	19

a Data (%) represent the mean of biologically independent samples (*n*=3). Only N1 is shown (*n*=1), and only N3 is shown (*n*=2).

b S represents the sulfur-oxidizing bacterium.

c nd represents not determined.

**Table 2:** Average relative taxonomic distribution of *nosZ* gene reads within the *Bacteroidetes* lineage at the genus level.

Genus of the lowest E-value (Accession numbers)	Class^b^	%^a^							

		N3	N2	N1	Ni	Ei	E1	E2	E3
unclassified*Flavobacteriales* (EDP71844)	F	0	5	0	7	11	6	4	19
unclassified*Flavobacteriaceae* (PCI11421)	F	6	13	5	14	10	17	5	7
*Lutibacter* spp. (WP_090224353, AMC12244, AFX81533, KUO67356)	F	7	8	29	20	10	12	14	14
*Seonamhaeicola* spp. (WP_076698534)	F	0	0	0	0	6	2	4	0
*Maribacter* spp. (WP_079511925, EAR02377, APQ16066)	F	9	5	0	7	6	8	17	4
*Arenibacter* spp. (WP_072764458, GAU57464)	F	8	1	5	3	5	3	1	0
*Gaetbulibacter* sp. (WP_099565396)	F	2	7	5	2	5	1	4	9
*Aquimarina* spp. (EZH71920)	F	11	9	0	11	4	3	0	0
*Cellulophaga* spp. (WP_047414439, WP_084061063)	F	5	1	10	3	4	3	7	2
*Bizionia* spp. (EGV44024, OBX22568)	F	4	0	0	1	4	1	2	0
*Muricauda* spp. (WP_045802108, AEM71845)	F	8	3	0	2	4	3	2	0
*Vitellibacter* spp. (OAD90232, KXO01187)	F	3	0	10	2	3	0	1	2
*Xanthomarina* sp. (WP_007651736)	F	0	3	0	0	2	0	0	0
*Tenacibaculum soleae* (OCK42558)	F	0	12	0	0	2	1	2	5
*Zobellia galactanivorans* (CAZ96392)	F	0	0	5	0	2	6	0	2
*Algibacter alginicilyticus* (WP_054727401)	F	0	0	0	2	2	0	2	2
*Myroides* spp. (EKB05496, KZE78434, EHO10957, GAQ13346)	F	8	0	0	2	1	1	0	2
*Aequorivita sublithincola* (AFL79671)	F	2	0	0	0	1	0	4	0
*Flavobacterium enshiense* (ESU23366)	F	0	0	0	0	1	0	0	0
*Mangrovimonas yunxiaonensis* (KFB01940)	F	0	3	0	2	1	5	0	6
*Robiginitalea biformata* (EAR16321)	F	0	2	0	0	1	0	1	7
*Ulvibacter litoralis* (WP_093139708)	F	0	1	0	0	1	0	2	0
*Capnocytophaga* spp. (CEN36467, EGD34772, WP_095896667)	F	5	0	5	1	1	0	0	3
*Psychroflexus gondwanensis* (EMY80080)	F	0	0	0	0	1	1	0	7
*Gelidibacter algens* (OBX25598)	F	0	0	0	0	0	1	0	0
*Gramella forsetii* (CAL66385)	F	0	0	0	0	0	1	0	5
*Imtechella halotolerans* (EID76831)	F	3	1	0	1	0	5	0	0
*Owenweeksia hongkongensis* (AEV34412)	F	2	0	0	0	0	3	0	0
*Salegentibacter mishustinae* (KRG30563)	F	3	1	0	0	0	2	2	0
*Zhouia amylolytica* (ETN94445)	F	0	3	0	1	0	1	0	0
*Fulvivirga imtechensis* (ELR70764)	Cy	6	7	0	1	1	1	2	0
*Cesiribacter andamanensis* (EMR03384)	Cy	0	0	5	0	0	0	0	0
*Rufibacter* spp. (AMM52593, AKQ46153)	Cy	0	0	0	2	0	0	4	4
*Runella slithyformis* (AEI46569)	Cy	0	0	0	0	0	0	2	0
*Haliscomenobacter hydrossis* (AEE53312)	Sa	0	0	0	0	1	4	0	0
*Phaeodactylibacter xiamenensis* (KGE85855)	Sa	3	2	0	1	2	1	0	0
*Pedobacter glucosidilyticus* (KHJ39550)	Sh	0	0	0	1	0	0	0	0
*Chlorobium* spp. (KXB98523, KXK48569)	Ch	2	0	5	0	1	0	1	0
unclassified bacterium	nd	6	12	19	13	6	8	18	0

a Data (%) represent the mean of biologically independent samples (*n*=3). Only N1 is shown (*n*=1), and only N3 is shown (*n*=2).

b F, *Flavobacteriia*; Ch, *Chlorobia*; Cy, *Cytophagia*; Sa, *Saprospiria*; Sh, *Sphingobacteriia*.
