# Supplementary material for: Eelgrass Sediment Microbiome as a Nitrous Oxide Sink in Brackish Lake Akkeshi, Japan
Source: Microbes Environ. 2018 Dec 1;34(1):13–22. doi: 10.1264/jsme2.ME18103 (PMC6440730; doi:10.1264/jsme2.ME18103)
Supplement: Supplementary file 1 [file 34_13_s1.pdf]

Supplementary information

Materials and Methods

Amplification of nosZ *genes clade II, cloning, sequencing, and phylogenetic analysis*

Partial *nosZ* gene fragments were amplified from single extracted DNA E3 sediment with TaKaRa *EX Taq* DNA polymerase (Takara Bio, Japana). The total volume of PCR mixture was 50 µL including 5 µL of 10×*EX Taq* buffer, 250 µM each of deoxynucleoside triphosphate, 0.5 µM of each primer, and 1.25 U of *EX Taq* DNA polymerase. The primer sets were and the modified nosZ-II-Fn (5′-CTN GGN CCN YTK CAY AC-3′) and nosZ-II-Rn (5′-GCN GAR CAR AAN TCB GTR C-3′) for *nosZ* gene clade II (29). The following protocol was used for *nosZ* gene clade II: an initial denaturation step at 95°C for 5 min and then 35 cycles at 95°C for 30 s, 54°C for 30 s and 72°C for 60 s, with a final 10-min extension at 72°C. After electrophoresis, the amplified gene fragments were cloned into the vector pCR2.1-TOPO with a TOPO TA PCR cloning kit (Life Technologies, Carlsbad, CA, USA). White colonies were grown overnight at 37°C on fresh LB broth agar plates with kanamycin. The inserts were amplified by direct PCR from a single colony with the vector-specific primers M13F and M13R. After PCR products were electrophoresed again in an agarose gel to check the length of the bands, amplicons were purified with a QIAquick PCR purification kit (QIAGEN, Valencia, CA, USA) prior to sequencing. Both strands of M13F/M13R PCR products were sequenced with a BigDye Terminator version 3.1 (Life Technologies) on a 3130x1 Genetic Analyzer (Life Technologies). A maximum-likelihood phylogenetic tree was constructed based on alignments of *nosZ* amino acid

sequences using MEGA7. Bootstrap values were determined from 500 replications.

Chemical analysis

Ten milliliters of surface water and the pore water of sediment were filtered using Millex-GP (pore size, 0.2 μm, Thermo Fisher Scientific, Waltham, MA, USA) to remove cells from the water right after the sampling. The samples were transferred to the laboratory in an ice-cooled box within 3 days. The concentrations of nitrate and sulfate in the surface water and the pore water of sediment were measured with an ion chromatography system-1000 attached to an UltiMate 3000 Variable Wavelength Detector (Dionex, Thermo Fisher Scientific, Sunnyvale, CA).

Blastx analyses for norB *and* amoA *genes*

To detect nitric oxide reductase subunit B (*norB*) and *amoA* gene reads in the metagenomes derived from each sediment sample, publicly available NorB and AmoA amino acid references were downloaded from the FunGene (http://fungene.cme.msu.edu) of the Ribosomal Database Project (RDP) and the National Center for Biotechnology Information (NCBI), and then imported into CLC Genomic Workbench. NorB or AmoA -encoding reads were identified by blastx against the NosZ or AmoA amino acid references with an e-value threshold of 10^{-15}.

Nucleotide sequence accession numbers

The *nosZ* gene sequences obtained from the Sanger sequencer were submitted to the DDBJ/EMBL/GenBank and have been assigned the following accession numbers: LC384881 to LC384891.

Supplementary Table S1

Table S1. Numbers of reads used for blastx analysis, read numbers of *nosZ, norB, and amoA* genes, and its abundance ratios.

Sample name	Biological duplication	Sample ID	Number of read more than 90 of nucleotides length	Number of read used for blastx analysis	Numbr of *nosZ* gene read	Numbr of *norB* gene read	Numbr of *amoA* gene read	*nosZ* : *norB*
Ni	3	bN1	1,450,457	1,450,457	146	50	n.d.	1.0 : 0.3
		bN2	836,267	836,267	90	43	4	1.0 : 0.5
		bN3	541,421	541,421	67	27	n.d.	1.0 : 0.4
N1	1	aN4	4,494,965	1,349,140	115	59	n.d.	1.0 : 0.5
N2	3	aN1	3,333,749	1,005,525	96	35	n.d.	1.0 : 0.4
		aN2	2,455,516	736,655	87	45	1	1.0 : 0.3
		aN3	3,648,228	1,094,468	120	38	n.d.	1.0 : 0.5
N3	2	aN7	4,278,806	1,283,642	107	57	3	1.0 : 0.5
		aN9	3,510,408	1,053,122	96	48	1	1.0 : 0.5
Ei	3	bA1	1,684,435	1,684,435	164	47	n.d.	1.0 : 0.3
		bA2	1,000,872	1,000,872	93	46	n.d.	1.0 : 0.5
		bA3	1,567,272	1,567,272	151	76	n.d.	1.0 : 0.5
E1	3	aA4	1,123,649	1,123,649	75	77	n.d.	1.0 : 1.0
		aA5	1,285,441	1,285,441	101	59	n.d.	1.0 : 0.6
		aA6	1,640,282	1,640,282	146	55	n.d.	1.0 : 0.4
E2	3	aA1	1,153,169	1,153,169	77	49	n.d.	1.0 : 0.6
		aA2	1,186,662	1,186,662	81	56	n.d.	1.0 : 0.7
		aA3	892,224	892,224	52	37	n.d.	1.0 : 0.7
E3	3	aA7	979,482	979,482	77	67	n.d.	1.0 : 0.9
		aA8	563,074	563,074	23	23	n.d.	1.0 : 1.0
		aA9	402,760	402,760	22	29	n.d.	1.0 : 1.3
Total	21		38,029,139	22,830,019	1,986	1,023	9	

n.d. shows not detected.

Supplementary figure legends

Fig. S1. Sampling sites, the non-eelgrass zone (**A**) and eelgrass zone (**B**). The bottoms of the non-eelgrass zone (**C**) and eelgrass zone (**D**). The sediment sampled by coring in eelgrass zone (**E**). The 0.0–4.0 cm layer of sediments used for a laboratory incubation obtained from the non-eelgrass zone (**F**), and eelgrass zone (**G**).

Fig. S2. The phylogenetic relationship among 11 of amino acid sequences of *nozZ* gene clade II obtained from eelgrass sediment microcosms (E3) by using PCR cloning analysis and previously reported 138 of NosZ amino acid sequences. Bold type indicates *nosZ* clones derived from microcosms. The scale bar represents an estimated sequence divergence of 20%. Branches with bootstrap support more than 70% are revealed by closed circles.

Fig. S3. Rarefaction analysis of *nosZ* gene reads in *in situ* sediments and bottles incubated after 7 days in the non-eelgrass and eelgrass zones at lineages level. Unidentified environmental Operational taxonomic units (OTU) were defined by the lineages shown in Figure 3. Only N1 is shown (*n*=1), and only N3 is shown (*n*=2).

Fig. S4. Correlation between the increased concentration of N_2O in headspace of bottles and increased copy numbers of *amoA* genes after incubation in the non-eelgrass and eelgrass zones. (**A**) Sediments incubated without NH_4Cl or N_2O additions in the non-eelgrass zone N1 and eelgrass zone E1. (**B**) Sediments incubated with NH_4Cl addition in the non-eelgrass zone N2 and eelgrass zone E2.

Fig. S1

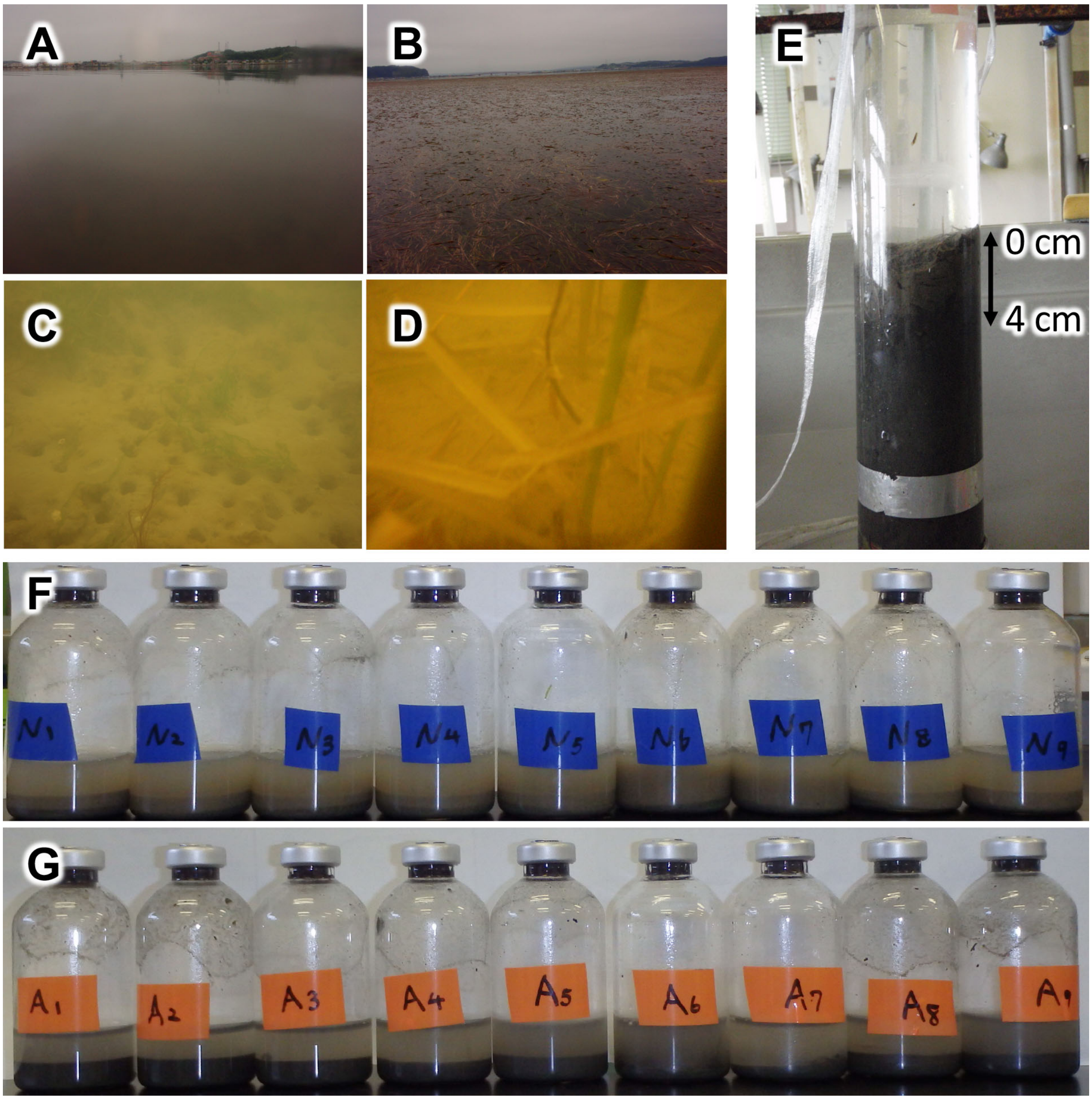

Fig. S1. Nakagawa *et al*.

Fig. S2

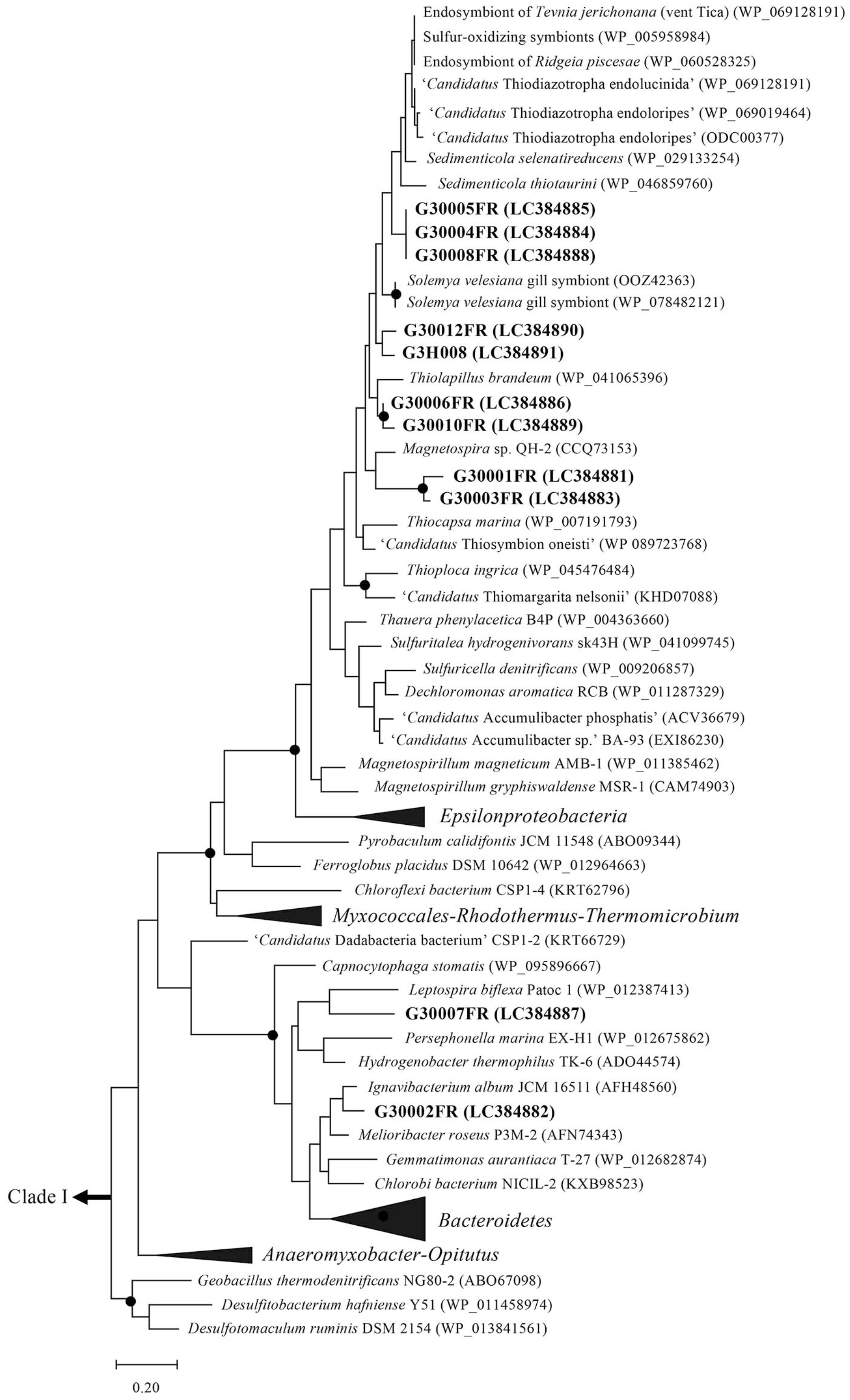

Fig. S2. Nakagawa *et al*.

Fig. S3.

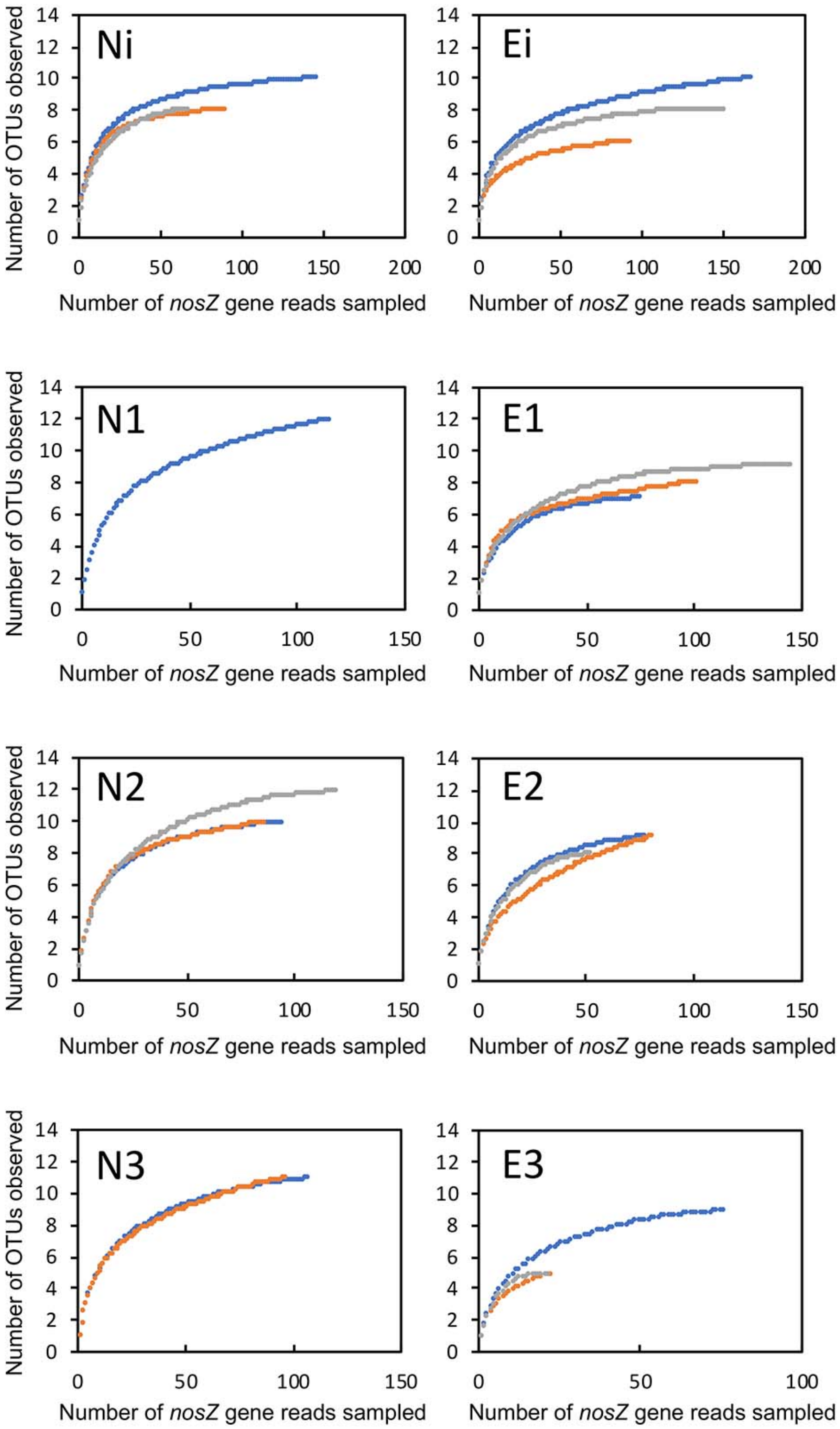

Fig. S3. Nakagawa *et al*.

Fig. S4.

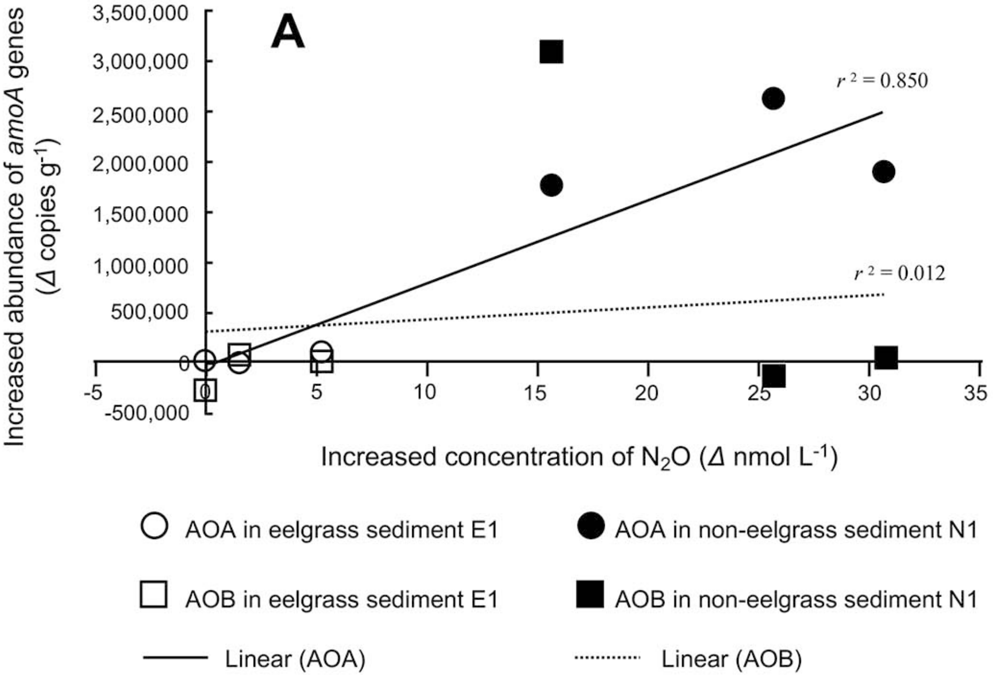

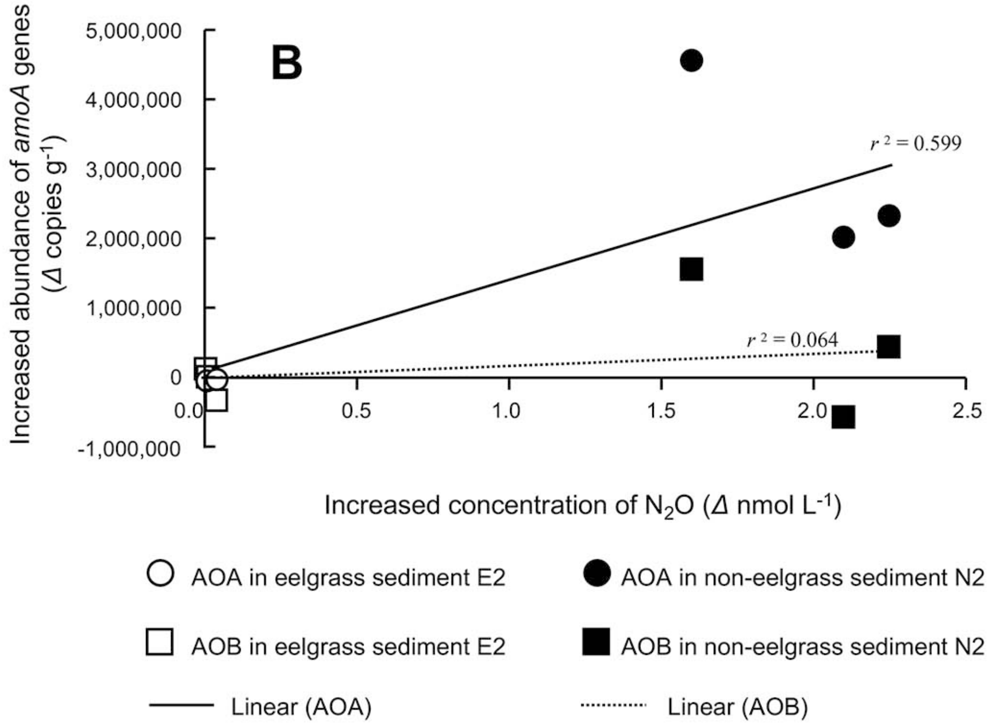

Fig. S4. Nakagawa *et al*.
